# Invasive meningococcal B disease: Spatiotemporal cluster identification using finetype data, the Netherlands, 2005–2023

**DOI:** 10.1017/S0950268826101368

**Published:** 2026-04-06

**Authors:** Marta Bertran, Jan van de Kassteele, Linda J. Visser, Mirjam J. Knol, Hester E. de Melker, Nina M. van Sorge, Anneke Steens

**Affiliations:** 1 Centre for Infectious Disease Control, National Institute for Public Health and the Environment, Netherlands; 2 European Programme for Intervention Epidemiology Training (EPIET), European Centre for Disease Prevention and Control, Sweden; 3 Amsterdam Institute for Immunology and Infectious Diseases, Amsterdam UMC Location AMC, Netherlands; 4 Netherlands Reference Laboratory for Bacterial Meningitis (NRLBM), Amsterdam UMC Location AMC, Netherlands

**Keywords:** meningococcal disease serogroup B, Neisseria meningitidis, finetyping, clusters, SaTScan

## Abstract

This study assessed whether systematically using finetype data in national surveillance of invasive meningococcal disease serogroup B (IMD-B) in the Netherlands could improve cluster detection in order to prevent further cases through public health actions. We analysed 2005–2023 data, including 1,642 IMD-B cases with complete finetype and municipality information (95%; *N* = 1729). Using a generalized linear model, we calculated expected baselines for each finetype, including temporal trends. Using SaTScan™, we applied Poisson scan-statistics with a 365-day window to identify spatiotemporal clusters, comparing results to epidemiological and core-genome multi-locus sequence typing (cgMLST) data. Of 453 finetypes, 308 (68%) occurred once; diversity was high (Gini-Simpson index 0.96). We identified 42 spatiotemporal clusters across 37 finetypes, comprising 132 cases (8%), with a median cluster size of two (range 2–21) and duration of 45 days (range 6–356). Between zero and five clusters were detected yearly. Among 18 cases with known epidemiological links, 14 (78%) were within detected spatiotemporal clusters. CgMLST data from eight clusters supported some clusters but rejected others. Systematic cluster detection using finetype could reveal missed epidemiological links, potentially enabling public health action. However, its impact in preventing additional IMD-B cases is likely limited due to small cluster sizes, though meaningful given the severity of IMD-B. Simple finetype mapping may provide a resource-efficient alternative to SaTScan™.

## Key results

- Of 1,642 IMD-B cases with complete finetype, there were 453 distinct finetypes, with 68% occurring only once.
- Strain diversity was high, with a 4% (annual range 3–9%) probability that two random cases shared the same finetype.
- Using SaTScan™, we detected 42 spatiotemporal clusters across 37 finetypes, accounting for 8% of cases, but most clusters were small (median size 2).
- Among 14 case-pairs present in the IMD-B data with confirmed epidemiological links, all 14 were captured within clusters detected by SaTScan™, indicating high sensitivity.
- Systematic cluster detection using finetype data is unlikely to prevent many additional cases due to small cluster sizes, but may help prioritize clusters for further epidemiological and/or genomic investigation, with simple finetype mapping offering a practical alternative to SaTScan™.

## Introduction

The Gram-negative bacterium *Neisseria meningitidis* can be carried asymptomatically or cause severe invasive meningococcal disease (IMD), such as meningitis and/or sepsis [1]. Disease progression can be very fast, with high case-fatality (5–10%) and severe long-term sequelae in around 6% of survivors [2]. Of the 12 identified serogroups, serogroup B has been the most prevalent in the Netherlands since surveillance began, and it remained so after the introduction of meningococcal C (2002) and ACWY (2018) vaccines in the National Immunization Programme (NIP) [3]. In 2022, after a decline during the COVID-19 pandemic, IMD incidence returned to pre-pandemic levels, with 88% of cases due to serogroup B [4]. Routine vaccination against serogroup B has not been implemented in the NIP.

*N. meningitidis* transmission occurs via respiratory droplets or secretions and requires prolonged or intense contact. The highest risk of secondary cases is within the first week following symptom onset of an index case, but remains elevated up to four weeks [5]. In the Netherlands, the Municipal Public Health Services (MPHS) conduct contact tracing and recommend chemoprophylaxis for close contacts to prevent secondary IMD and eliminate asymptomatic carriage [5]. Broader prophylaxis is recommended if at least two IMD cases occur in the same school class or daycare group within 31 days [5, 6]. Vaccination of contacts is recommended for unvaccinated persons exposed to serogroup A, C, W, or Y, but for serogroup B, it is limited to high-risk individuals (e.g. asplenia or complement deficiency). As licensed meningococcal B (menB) vaccines do not impact carriage (and therefore transmission), and clusters are considered rare [5–7], vaccination is not routinely recommended in case of IMD-B clusters.

In 2007, *porA* and *fetA* finetyping was internationally recommended for rapid investigation of meningococcal outbreaks [8]. This DNA sequence-based typing of variable regions (VR) of two *N. meningitidis* outer membrane proteins (PorA and FetA) has the advantage of being reproducible and portable, with most isolates being typeable, including non-cultured ones [8, 9]. In the Netherlands, *porA* and *fetA* finetyping of IMD isolates has been performed since 2005. However, cluster identification remains primarily based on epidemiological information and conducted by the MPHS [6]. At the National Institute of Public Health and the Environment (RIVM), finetype data are explored during routine surveillance but not systematically used for cluster identification, partly because the background probability that two cases with the same finetype are close in space and time is not well known.

In contrast, other countries have implemented molecular approaches in cluster detection. Germany, for example, implemented spatiotemporal analysis of IMD cases using finetype data and SaTScan™ in routine national surveillance [9, 10]. In France, cultured isolates and samples are systematically sent for full phenotyping and genotyping, including whole genome sequencing (WGS) [11]. In England, WGS is now used to investigate potential clusters and inform public health actions [12]. A 2011 study in England comparing finetype with other molecular methods (multi-locus sequence type (MLST), Factor H binding protein typing), found that finetype was still useful to rule out or support epidemiological links between cases close in space and time [13]. In the Netherlands, WGS is conducted in batches or ad hoc if a cluster is suspected, but costs and logistical constraints remain a barrier to implementing WGS for timely surveillance.

A better understanding of the predominant finetypes in the Netherlands may inform surveillance and aid in assessing the significance of potential clusters based on finetype. We aimed to assess whether systematically using finetype data could improve cluster identification and potentially prevent cases through public health actions, such as vaccination or chemoprophylaxis. Given the recent IMD increase and the dominance (nearly 90%) of serogroup B cases in the absence of routine vaccination, we focused on IMD-B. Our objectives were to describe IMD-B isolate diversity by finetype, identify the most common finetypes, and identify spatiotemporal IMD-B clusters by finetype using 2005–2023 Dutch surveillance data. We aimed to validate the identified clusters with epidemiological and WGS data.

## Methods

### Data sources

IMD was defined as the identification of *N. meningitidis* in a normally sterile site (e.g., blood, cerebrospinal fluid (CSF)) by culture or PCR in case of non-cultured samples. All Dutch medical microbiological laboratories voluntarily submit invasive meningococcal isolates or CSF/blood samples to the National Reference Laboratory for Bacterial Meningitis (NRLBM, Amsterdam UMC, Amsterdam, Netherlands). The NRLBM performs serogrouping using Ouchterlony gel diffusion on isolates, or, since 2015/16, by meningococcal-specific and group-specific real-time PCR (targeting the ctrA gene) on CSF samples, as previously described [14]. Finetyping by Sanger sequencing of *porA* and *fetA* has been routinely performed on isolates of all IMD cases received at NRLBM since 2005 and is attempted on culture-negative samples [8].

As IMD is a notifiable disease, clinicians and laboratories must report cases to the MPHS, who notify the RIVM. Notifications include demographic, clinical, and epidemiological data, including onset date, sample collection date, patient residence details, potential sources/places of infection, and any known epidemiological links. NRLBM laboratory data are routinely linked to notification data, comprising the national surveillance dataset [14]. On January 30, 2024, we extracted data on IMD-B cases reported between 01 January 2005 and 31 December 2023. Because we limited our study to IMD-B, cases without an NRLBM-typed sample/isolate were excluded; 97% of notifications during the study period had serogroup information.

We extracted annual population data by age as of January 1^st^ each year and annual postcode-level population estimates from Statistics Netherlands (CBS) [15]. For comparison with core-genome MLST (cgMLST) data, we used publicly available WGS data from pubMLST [16].

### Definitions

Index date was defined as the onset date, or if unavailable, the earliest of the sample date or NRLBM receipt date. In cases where the onset date was implausible given other known dates (*n = 8*), index dates were imputed based on other available dates.

Finetype was considered complete if results were available for *porA* VR1 and VR2 and *fetA* VR (including deletions). Incompleteness was due to a lack of material at the NRLBM or unreliable/no results, likely due to limited DNA quantities.

Municipality was assigned according to the patient’s residential postcode by matching the 4-digit postcodes to the 2023 municipalities. We assumed postal boxes corresponded to the municipality of residence. Cases where the postcode did not match official postcode lists were not assigned a municipality.

Age was categorized as <5, 5–14, 15–24, 25–64, and ≥ 65 years for descriptive analyses, and grouped further into <5, 5–24, 25–64, and ≥ 65 years to compare missing finetype data, given the small numbers. Years were grouped as 2005–2009, 2010–2014, 2015–2019, and 2020–2023 to increase the power to identify time trends.

We classified epidemiological links reported by the MPHS on notifications as I) confirmed, when the related case(s) were mentioned in the notification, II) probable, when the link described was in a very specific context (e.g. school) or, III) possible, if the notification stated in a generic context that the case was related to another, without specifying which case (e.g. friend in group).

### Analyses

We conducted all analyses in R (v4.4.3), except the cluster detection, which was done in SaTScan™ (v9.4.1) software. For categorical variables, we assessed differences in proportions using the chi-square or Fisher’s exact test in cases of small numbers. For continuous variables, we calculated medians and interquartile ranges (IQR), and assessed differences using the Wilcoxon Rank Sum test. We estimated overall and annual diversity using the Gini Simpson index (1-Simpson diversity index), which increases as diversity increases, using the R vegan package [17, 18].

For the spatiotemporal analysis, we excluded cases with incomplete finetype data or missing municipality and aggregated data by municipality. We excluded finetypes occurring once or twice with ≥2 years between the index date of cases. SaTScan™ computed an expectation-based Poisson scan statistic to identify clusters for each finetype [19]. The expected baseline included a linear time trend to account for the overall decreasing incidence during the study period. SaTScan™ uses scanning windows, which are cylinders for spatiotemporal analyses; the cylinder’s circular base represents space, and height represents time. Scanning windows originate at defined locations (in our case, municipality centroids), time points (index week), and increase in size (in space and time) [19]. The observed and expected case numbers inside the scan window are compared to numbers outside the window using a likelihood ratio test to detect clusters that are least likely to have occurred by chance. The window with the maximum likelihood is defined as the most likely cluster. The statistical significance for each cluster is obtained through Monte Carlo hypothesis testing (999 replications). Given confirmation of a 9-month cluster in a Belgian nursery in 2018 [20], we applied a maximum temporal window of 365 days to remain sensitive for long clusters that may have been missed in epidemiological investigations. We applied a maximum spatial window of 30% of the at-risk population, instead of 50% default. We considered this appropriate as the Dutch population is relatively mobile, with a considerable number of people commuting relatively far for work. We restricted the analysis to non-overlapping clusters.

We compared the spatiotemporal clusters detected with SaTScan™ with the epidemiological links identified by the MHPS.

### Genomic analysis

To validate some of the spatiotemporal finetype clusters detected with SaTScan™, we compared them with publicly available cgMLST data from PubMLST [16]. For clustered cases, we searched the isolates in PubMLST [16]. Where ≥2 isolates within a cluster were available, we used the genome comparator function to perform a pairwise comparison of those isolates within each cluster using the cgMLST(v3) scheme from the Bacterial Isolate Genome Sequence Database (BIGSdb) and assessed allelic differences (AD) between the pairs [16]. We also created a minimum spanning tree using GrapeTree [21]. We considered ≤25 AD between two isolates to support clustering and any AD>100 to reject clustering. For cases containing between 25 and 100 AD, we considered the time between their index dates, allowing for larger AD as the time interval increased to support clustering. For example, we considered an interval of 80 AD in a cluster lasting 321 days to be supported by WGS, but a cluster lasting 13 days with a 47 AD to be rejected by WGS.

## Results

**Figure 1.**
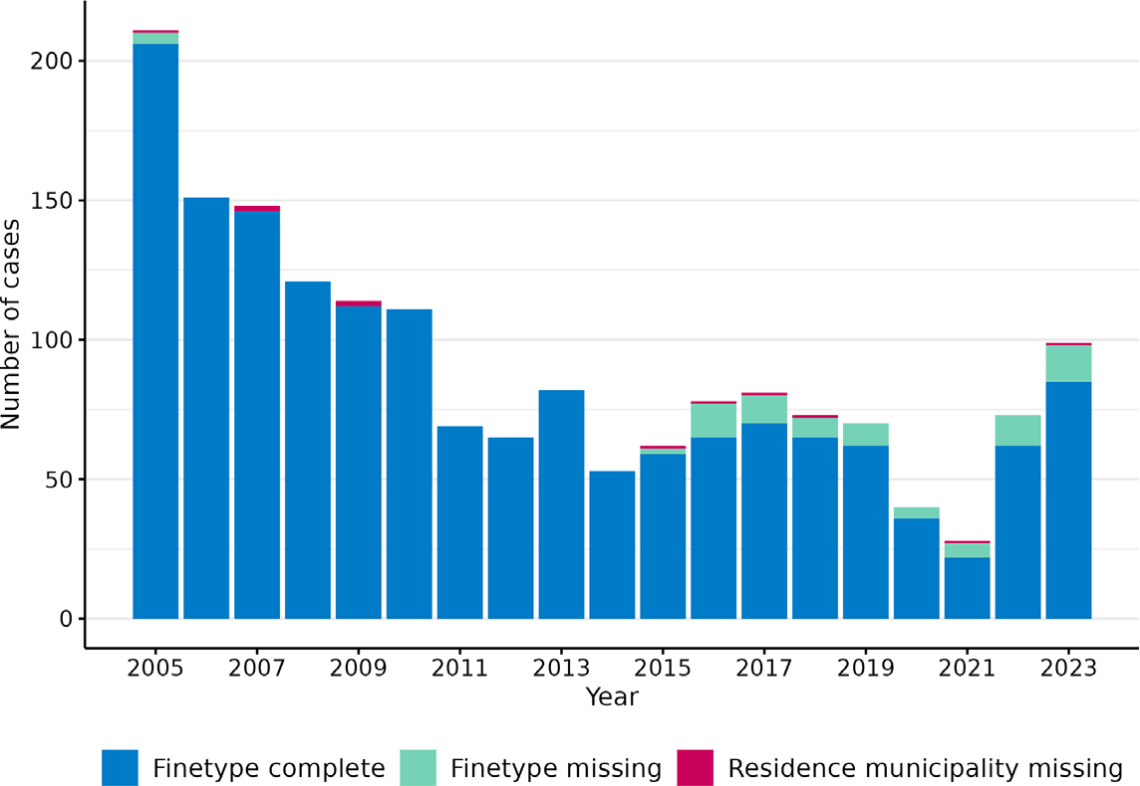
Number of annual IMD-B cases by inclusion criteria in spatiotemporal analysis, 2005–2023, Netherlands.

Overall, there were 1729 IMD-B reported cases with an index date between 2005 and 2023 (annual range 28–211). The number of IMD-B cases followed a decreasing trend from 2005 to 2021, oscillating between 2013 and 2017, and with a large drop in 2020 and 2021 related to the COVID-19 pandemic social restrictions (Figure 1). Post-COVID-19, the number of cases increased in 2022 and 2023, reaching its highest peak since 2011 (*n = 99*; incidence = 0.55 per 100,000 population). The median age of cases was nine (IQR 1–23) years, with 42% of cases occurring in <5-year-olds (*n = 724*), followed by 15–24-year-olds (*n = 360*; 21%). Among cases with sex data (*n = 1679*), 49% were females (*n = 816*) (Table 1).

**Table 1.** Finetype completeness of IMD-B cases by different characteristics, 2005–2023, The Netherlands

Variable	Category	Overall(*N* = 1729)			Finetype complete(*N* = 1653)			Finetype incomplete(*N* = 76)			*p-*value *_a_*
		*n*	*N*	*%*	*n*	*N*	*%*	*n*	*N*	*%*	
Sex	Female	816	1,679	49	778	1605	48	38	74	51	0.6
	Male	863	1,679	51	827	1605	52	36	74	49	
Age group (years)	0–4	724	1728	42	702	1652	42	22	76	29	0.020
	5–24	587	1728	34	549	1652	33	38	76	50	
	25–64	263	1728	15	254	1652	15	9	76	12	
	≥65	154	1728	8.9	147	1652	8.9	7	76	9.2	
Period	2005–2009	745	1729	43	741	1653	45	4	76	5.3	<0.001
	2010–2014	380	1729	22	380	1653	23	0	76	0	
	2015–2019	364	1729	21	325	1653	20	39	76	51	
	2020–2023	240	1729	14	207	1653	13	33	76	43	
Sample type	Blood isolate	846	1723	49	839	1647	51	7	76	9.2	<0.001
	CSF isolate	735	1723	43	732	1647	44	3	76	3.9	0.6
	Blood/CSF (PCR)^b^	125	1723	7.3	61	1647	3.7	64^b^	76	84	
	Other invasive material isolate	17	1723	1.0	15	1647	0.9	2	76	2.6	0.020

*Note: CSF, Cerebrospinal fluid; PCR, Polymerase Chain Reaction.*

a Pearson’s Chi-squared test; Fisher’s exact test;

b 61/64 PCR samples were CSF samples.

Of the 1729 cases, 96% (*n = 1653*) had complete finetype data, comprising 454 distinct finetypes. Eleven (0.7%) additional cases lacked information on municipality of residence, leaving 1,642 for spatiotemporal analysis. The annual proportion of isolates with complete finetype ranged from 82% to 100%, but decreased over time, especially since 2016 (Figure 1). This decrease was associated with an increased number of non-cultured CSF samples submitted for serogrouping, which accounted for 80% (61/76) of cases without finetype data (Table 1). Of all CSF-positive cases (culture/PCR), cases with non-cultured CSF samples increased from 0% in 2005–2014, to 25% (46/181) in 2015–2019, and 54% (72/133) in 2020–2023. Out of all cases, this proportion was 13% (46/364) in 2015–2019 and 30% (72/240) in 2020–2023.

**Figure 2.**
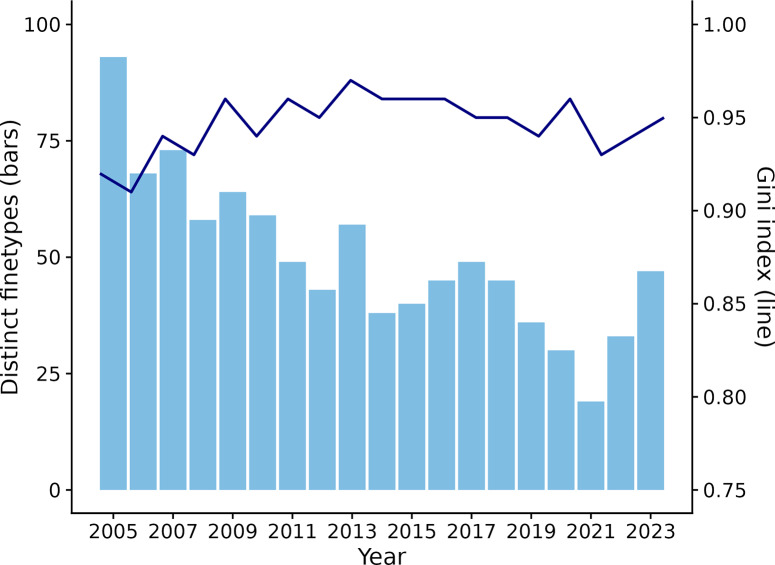
Number of distinct finetypes (bars) and Gini diversity index (line) by year.

The number of distinct finetypes annually followed a similar trend to the number of annual cases (Figure 2). The overall diversity index was 0.96, and the annual diversity ranged from 0.91 in 2006 to 0.97 in 2013, indicating that the probability of two randomly selected cases being caused by the same finetype was 4% (range 3–9%) (Figure 2).

Among the 1,642 cases with complete data, we identified 453 distinct finetypes; 308 (68%) occurred once and 28 (6%) twice with a ≥ 2-year interval between the index date of cases. The remaining 117 (26%) finetypes were included in the spatiotemporal cluster analysis and accounted for 1,278 cases (74% of cases). These 117 finetypes caused a median of 5 (IQR 3–9) cases during the entire study period, and 62% (72/117) caused 2–5 cases.

**Figure 3.**
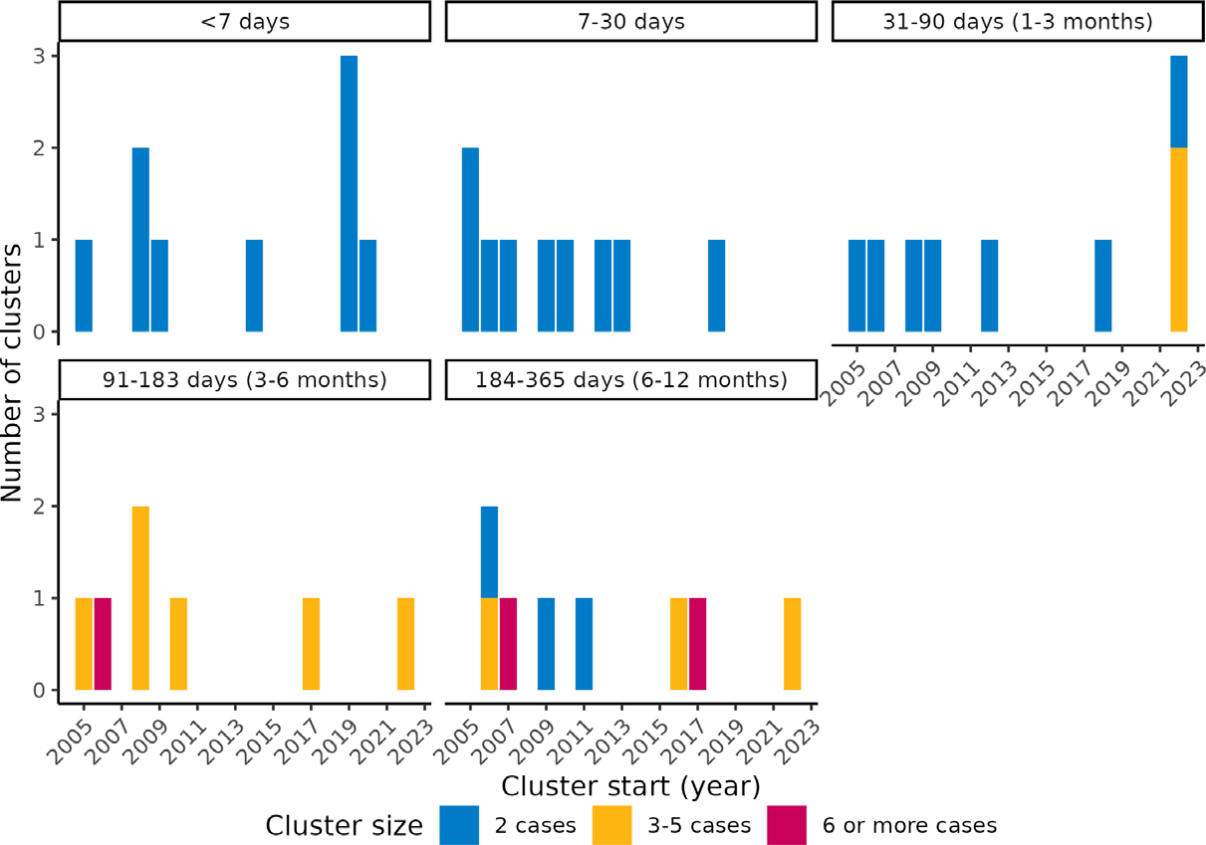
Number of IMD-B spatiotemporal finetype clusters by year (x-axes), duration (sub-plots) and number of cases (colors), The Netherlands, 2005–2023.

We identified 42 spatiotemporal clusters across 37 finetypes, which accounted for 32% (37/117) of finetypes tested and 8% (37/454) of all IMD-B finetypes in the study period (Table 2). The number of clustered cases accounted for 10% (132/1278) of cases included in the cluster analysis and 8% (132/1729) overall. There were between 0 and 5 clusters per year, with no temporal trend (Figure 3). The median number of clusters per finetype was one (range 1–3), with a median size of two (IQR 2–3; range 2–21) cases per cluster and a median cluster duration of 45 (IQR 13–158) days. Cluster duration ranged from 6 to 356 days, but 64% (*n = 27*) of clusters lasted ≤3 months (91 days), of which all but two consisted of two cases. Of the clustered cases, 33% (*n = 43*) occurred at least 21 days after the second case of the respective cluster, a time frame in which, hypothetically, vaccination could have potentially prevented the disease, if offered and eligible. Among the clustered cases, 83% (*n = 109*) were < 25-years-old (39% <5 years and 44% 5–24-years-old), compared with 76% (*n = 1,311*) among all cases.

**Table 2.** Characteristics of IMD-B clusters detected with SaTScan™, including epidemiological and whole-genome sequencing information

Finetype	Total cases	Percent of total	Number of clusters	Clustered cases	Cluster start (year)	Cluster duration (days)	Median age (years)	Age range (years)	Number of MPHS	Epidemiological link	Setting	Cases with cgMLST data	Allelic distance range	cgMLST supports clustering?
P1.7–2,4:F1–5	244	14.1%	1	21	2007	286	6	0–83	3	Confirmed (n = 4); Probable (n = 1)	Nursery/School [3]; General (friends) [2]	3	110–577	Rejects
P1.5–2,10:F5–1	86	5.0%	3	9	2006	174	4	0–19	3	Unknown	N/A	3	37–179	Uncertain (n = 2); Rejects clustering to 3^rd^ isolate
				3	2008	132	4	3–5	1	Unknown	N/A	0	–	–
				2	2009	6	6	6–6	1	Confirmed (n = 2)	Nursery/School	0	–	–
P1.22,14:F5–1	49	2.8%	2	2	2014	6	6	5–7	1	Probable (n = 2)	Family/ household	2	35	Rejects
				6	2017	321	18	13–59	1	Unknown	N/A	6	9–80	Supports
P1.22,9:F5–12	40	2.3%	2	5	2022	90	15,5	14–20	2	Possible (n = 2)	General (friends)	0	–	–
				3	2022	41	40	18–58	1	Unknown	N/A	0	–	–
P1.19,15:F5–1	29	1.7%	1	5	2016	279	1	0–69	2	Unknown	N/A	0	–	–
P1.22,14:F1–7	26	1.5%	1	4	2010	160	38,5	0–84	3	Unknown	N/A	1	–	–
P1.7–2,16:F3–3	24	1.4%	2	3	2005	146	0	0–8	2	Unknown	N/A	0	–	–
				2	2019	6	43,5	17–70	1	Unknown	N/A	1	–	–
P1.7,16–32:F3–3	21	1.2%	1	2	2007	13	3	3–3	1	Confirmed (n = 2)	General (friends)	0	–	–
P1.21,16:F1–15	15	0.9%	1	2	2006	27	2,5	0–5	1	Unknown	N/A	0	–	–
P1.7–1,1:F1–5	13	0.8%	1	3	2022	153	3	2–16	2	Unknown	N/A	0	–	–
P1.7–2,13–2:F4–1	12	0.7%	1	2	2010	20	6	4–8	2	Unknown	N/A	0	–	–
P1.18–1,30–3:F3–3	9	0.5%	1	2	2022	83	18	17–19	1	Possible (n = 2)	Family/ household	1	–	–
P1.22–1,14:F5–2	9	0.5%	1	2	2019	6	1,5	1–2	1	Possible (n = 2)	Family/ household	1	–	–
P1.5–2,10:F5–50	8	0.5%	1	2	2008	62	14	13–15	1	Unknown	N/A	0	–	–
P1.19–1,15–11:F3–7	7	0.4%	1	4	2006	328	9	4–46	2	Unknown	N/A	1	–	–
P1.19,15:F1–5	7	0.4%	1	2	2006	356	5,5	4–7	1	Unknown	N/A	0	–	–
P1.22,14–10:F1–5	7	0.4%	1	4	2008	160	3	3–5	1	Confirmed (n = 2); Probable (n = 1)	Nursery/ School	0	–	–
P1.7–2,13–2:F1–7	7	0.4%	1	2	2019	6	26	24–28	1	Unknown	N/A	0	–	–
P1.7–2,4:F4–3	7	0.4%	1	3	2017	146	26	0–69	3	Unknown	N/A	3	23–179	Supports clustering (n = 2); To third isolate rejects clustering
P1.22,9:F1–5	6	0.3%	1	2	2012	13	0,5	0–1	2	Unknown	N/A	2	47	Rejects
P1.22,14:F3–3	5	0.3%	1	3	2022	258	18	1–47	1	Unknown	N/A	0	–	–
P1.5–2,10–3:F5–1	5	0.3%	1	2	2008	6	10,5	9–12	1	Confirmed (n = 2)	General (friends)	0	–	–
P1.19,15:F3–3	4	0.2%	1	2	2018	13	16,5	15–18	2	Unknown	N/A	2	100	Rejects
P1.7–2,13–1:F4–1	4	0.2%	1	2	2005	8	2,5	1–4	1	Unknown	N/A	0	–	–
P1.7–2,16–32:F3–3	4	0.2%	1	2	2006	90	2,5	2–3	1	Unknown	N/A	0	–	–
P1.17,16–3:F1–65	3	0.2%	1	2	2005	62	5	5–5	1	Unknown	N/A	0	–	–
P1.19–1,3:F5–1	3	0.2%	1	2	2005	20	18	18–18	1	Unknown	N/A	0	–	–
P1.19–5,15:F5–1	3	0.2%	1	2	2012	48	34,5	16–53	2	Unknown	N/A	0	–	–
P1.19,15:F4–28	3	0.2%	1	2	2013	13	22	5–39	1	Probable (n = 2)	Family/ household	0	–	–
P1.7–2,13–1:F1–7	3	0.2%	1	2	2018	41	55	37–73	1	Unknown	N/A	1	–	–
P1.18–7,9:F1–5	2	0.1%	1	2	2005	6	5,5	5–6	1	Probable (n = 2)	Nursery/ School	0	–	–
P1.19,15:F1–20	2	0.1%	1	2	2011	286	0,5	0–1	2	Unknown	N/A	1	–	–
P1.22,10–1:F5–12	2	0.1%	1	2	2009	41	9	4–14	2	Unknown	N/A	0	–	–
P1.7–2,16:F3–6	2	0.1%	1	2	2020	6	2	0–4	1	Unknown	N/A	2	4	Supports
P1.7–2,16:F3–6	2	0.1%	1	2	2009	13	10	9–11	1	Possible (n = 2)	Nursery/ School	0	–	–
P1.7–2,4:F4–12	2	0.1%	1	2	2009	321	5,5	2–9	1	Unknown	N/A	0	–	–
P1.7,16–97:F3–3	2	0.1%	1	2	2008	6	3,5	2–5	1	Confirmed (n = 2)	Family/ household	0	–	–

**Figure 4.**
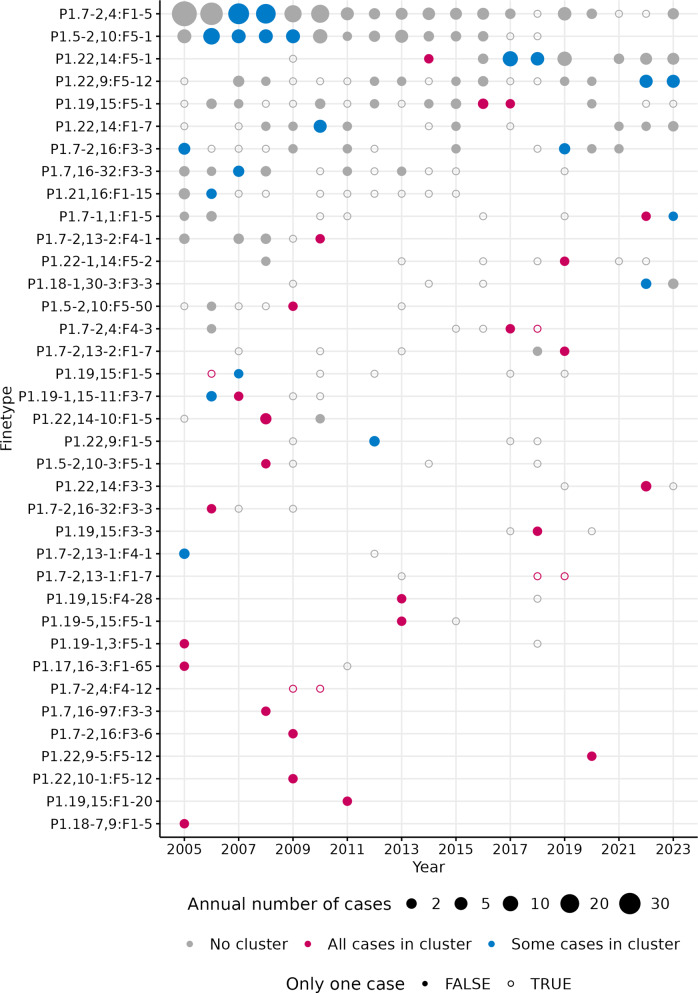
Timeline of annual IMD-B cases by finetype, for the 37 finetypes with spatiotemporal clusters. *Note*: ‘Some cases in cluster’ indicate that some of the cases for the respective finetype in that year were part of an identified cluster; ‘All cases in cluster’ indiciate that all cases for the respective finetype in that year were part of the cluster. A cluster may include cases from multiple years, but this is not directly indicated in the figure. ‘Only one case’ = TRUE indicates sporadic cases in that year for that finetype, but it may be within a cluster spanning multiple years.

Out of the ten most frequent finetypes, which accounted for 40% of all cases, eight finetypes were among the detected clusters. The second most common finetype (P1.22,14:F5–5; *n = 138*; 8% of total cases) was present throughout the whole study period, but was not identified as causing clusters. In contrast, 12 finetypes with ≤3 cases each were identified as causing clusters (Figure 4, Table 2).

**Figure 5.**
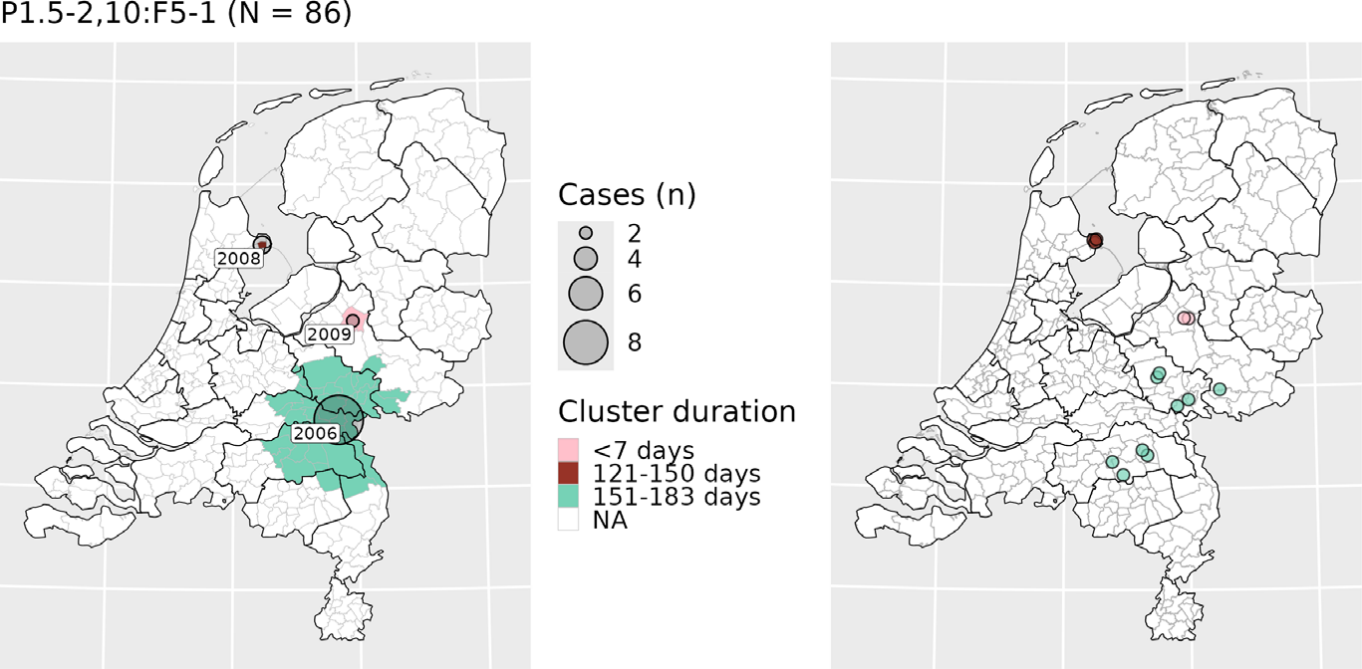
Maps showing clusters caused by finetype P1.5–2,10:F5–1 (left) and the location of the corresponding cases (right), The Netherlands, 2005–2023. Note: cases have been plotted using a jitter plot based on municipality of residence. The legend applies only to the left panel; colors of the cases in the right panel correspond to the colors of the clusters shown in the left panel.

Of clusters, 36% (*n = 15*) consisted of cases spread across multiple MPHS. An example of such a cluster is included in Figure 5, which shows the geographical distribution of potential clusters by the third most common finetype (P1.5–2,10:F5–1; *n = 86*) and the corresponding cases. There were 18 cases where an MPHS confirmed an epidemiological link between cases, of which 14 (78%) cases were part of the spatiotemporal clusters, distributed across six clusters. For the four cases not detected within spatiotemporal clusters, the paired links were missing from our dataset (not typed). The spatiotemporal analysis identified 16 additional cases that appeared to have a probable (*n = 8*) or possible (*n = 8*) epidemiological link, but the MPHS had not confirmed those. Within the clusters, the maximum number of cases with a confirmed or apparent epidemiological link was three (Table 2). In the largest detected cluster (*n = 21*), five cases had a confirmed epidemiological link, but these were identified as two subgroups: one with two cases and one with three cases. Sixteen other cases, not part of the identified spatiotemporal clusters, had a probable (*n = 3*) or possible (*n = 13*) epidemiological link reported by the MPHS.

CgMLST data for at least two cases were only available within eight of the 42 spatiotemporal clusters, accounting for 23 cases. This data supported some clusters (including a cluster lasting 356 days) but rejected others (including clusters lasting <7 days), without a clear pattern (Table 2). In some instances, where there appeared to be strong evidence of an epidemiological link between two cases within a short time period (<1 week), cgMLST data rejected clustering (AD>25). Conversely, cgMLST supported clustering in a previously reported community cluster lasting nearly a year (P1.22,14:F5–12017 cluster; AD range 9–80) where some, but not all, cases were directly/indirectly linked with a school (Table 2).

## Discussion

Using routinely collected finetype data in combination with SaTScan™, we retrospectively identified 42 IMD-B spatiotemporal clusters between 2005 and 2023. Of these, 36 clusters had no confirmed epidemiological links. The diversity of *N. meningitidis* strains causing IMD-B in the Netherlands was high, with a probability of two randomly selected cases being caused by the same finetype being lower than 10% in any given year. This diversity suggests finetype is valuable as a screening tool for IMD-B clusters in the Dutch context. However, it was difficult to establish whether the identified spatiotemporal clusters were true clusters, as we were unable to consistently validate these with epidemiological and/or cgMLST data. Most clusters were small and, in some cases, of long duration.

The small cluster size is in line with previous literature [7, 9, 22]. Although the identified size of a cluster may be influenced by the ability to confirm an epidemiological link beyond 2–3 cases in field studies, a space–time nearest neighbour study investigating IMD clustering in the Netherlands between 1993–2001 also concluded that clustering beyond chance only occurred among first-order nearest neighbours (i.e. only secondary cases) and not in larger clusters [7]. Similarly, in a spatiotemporal IMD-finetype study conducted in Germany using SaTScan™, 23/26 (88%) clusters consisted of 2–3 cases [9]. While that study used a 30-day maximum temporal window, in line with local cluster guidelines, we chose a wider timeframe for higher sensitivity because IMD-B clusters of prolonged duration have been previously reported [12, 20, 23, 24]. Our maximum cluster duration was 356 days, and 19% (8/42) of the identified clusters lasted ≥6 months. Nevertheless, our study still resulted in the same mean cluster size. Although some of the prolonged clusters we identified may be false positives, others were supported by cgMLST data, including one cluster of six cases lasting nearly a year. The time window used for cluster identification thus seems relevant. In Belgium, a cluster of three cases with a nine-month interval between the first and last case was reported in a nursery. Both cases had identical genotype and nearly identical cgMLST profile (P5–1, 2–2:F5–1; cc269) [20]. In England, a three-case cluster spanning 6 months was detected after isolates of two temporally close cases were identified as the same strain through WGS (P1.12–1,16–183) [12]. The identification of a genomic link prompted further epidemiological investigations, revealing an (indirect) epidemiological link and an earlier third case, with whom the links were previously not identified [12].

The potential added value of using systematic cluster detection based on finetype primarily lies in early identification of additional (potential) clusters that might go unnoticed by MPHS, particularly non-obvious epidemiological links between cases further apart in time, outside enclosed settings (i.e. household/school) or across multiple municipalities/MPHSs. In our study, among the cases with confirmed epidemiological links, all those present in the dataset were within the spatiotemporal clusters, suggesting the method was sensitive. All those clusters lasted <30 days and were mostly in enclosed settings (family/school/nursery). Still, there were seven spatiotemporal clusters lasting up to 3 months where the epidemiological information indicated a possible or probable epidemiological link. Unfortunately, except for one of these clusters, where cgMLST rejected clustering (35 AD), no cgMLST data were available for comparison.

SaTScan™ has been used for both retrospective and prospective identification of infectious disease clusters, including IMD [9, 10, 25]. While SaTScan™ detects statistically significant clusters without requiring manual review by public health officials or epidemiologists, implementation within routine surveillance would require dedicated resources, including time and programming skills. Given that IMD-B clusters were rare and small, plotting maps by finetype and time period within routine surveillance may yield similar results. The high finetype diversity among IMD-B strains indicates that two cases with the same finetype close in space and time should already trigger further epidemiological and/or genomic investigation(s) in order to enable public health action if needed. Our study’s finetype description can serve as a reference to determine whether this is a new or unusual finetype.

Nevertheless, the small cluster sizes suggest the overall potential public health benefits of broader cluster detection by finetype may be limited, as there are few potentially preventable cases. Furthermore, additional public health measures may be challenging to implement if clusters span over long periods or cases are scattered in non-specific community settings. Secondary cases who were close contacts should have already been identified through epidemiological investigations and would have been offered antibiotic prophylaxis. If vaccination was recommended for IMD-B clusters, as is done for serogroup A, C, Y or W IMD cases/clusters [5], a third of the clustered cases (*n = 43*) occurring ≥21 days after the second cluster case might have been prevented if they were vaccinated following the second case (assuming a two-week immunity build-up and eligibility). However, epidemiological information indicated that these later cases were rarely in settings where vaccination would be advised (such as nurseries/schools). Additionally, as currently licensed MenB vaccines do not impact carriage (unlike MenACWY vaccines), a vaccination policy for close contacts of an IMD-B case might still not prevent subsequent cases. Still, while the overall number of additional preventable cases may be limited, even preventing a small number of additional cases could be meaningful given the severity of IMD-B. Given the limitations of IMD-B vaccination for clustered cases and that 83% of clustered cases were in <25-year-olds (and 76% of all cases), implementing universal vaccination in the NIP would likely have a greater impact on preventing sporadic and clustered cases than adding vaccination to the IMD-B cluster guidelines.

The inclusion of nearly two decades of IMD-B national data, for which ~95% of cases had finetype and municipality data, was a strength of our study. While a larger proportion of the more recent cases lack finetype data due to PCR testing of culture-negative CSF samples, this approach has likely improved IMD case ascertainment compared to culture alone and may still enhance cluster identification, despite incomplete finetype information.

Our study had some limitations. Firstly, using the municipality of residence may not have been the most accurate geographical reference, as transmission can occur elsewhere, particularly given the high mobility of the Dutch working-age population. To account for this, we used a relatively large spatial window (30% of the population), compared to the ~7% used in a comparable analysis in Germany [9]. This enabled us to detect clusters spanning multiple MPHS regions, which would have likely been missed by local MPHS surveillance. However, as most IMD-B cases were in children, adolescents, and older adults, who are more likely to attend school near home or no longer commute for work, this limitation is unlikely to have had a large impact. Nevertheless, for 18–25-year-olds, who may study or work outside their residential municipality, some transmission links may have been missed. Secondly, two sporadic cases may be spuriously detected as a cluster, which may be enhanced by the large temporal and spatial windows parameters we chose and the small numbers per finetype. To use this method as an initial screening tool for further investigation, the balance between potential added value and additional workload should be considered. On the contrary, because the spatiotemporal analysis was based on an expected linear trend, finetypes which were common during the whole study period (such as P1.22,14:F5–5) had a lower chance of being detected as clusters and should be assessed cautiously. Finally, our ability to validate the detected clusters was limited by the cgMLST data available in PubMLST. For most clusters, either no strain or only a single strain was available, making it impossible to assess genetic relatedness for those clusters. Only in a small number of clusters were at least two isolates available for comparison, and even then, these sometimes represented just a fraction of the total cases within those clusters. For example, in the largest cluster detected, only 3/21 isolates could be analysed, meaning we cannot reliably confirm or reject clustering for the entire cluster based on this limited subset. Because such a large proportion of isolates lacked genomic data, we could not reliably estimate the sensitivity or positive predictive value of our cluster detection method, since the gold standard (WGS) was not available for most cases. Furthermore, there is no established consensus on the AD threshold to define an IMD-B cluster [26, 27]. While in the European Meningococcal Epidemiology in Real Time (EMERT) II project, a 10 AD distance is considered the limit to define a single-linkage cgMLST cluster, a retrospective US study comparing outbreak cases with cgMLST data found that previously reported IMD-B outbreaks had an intra-outbreak AD ranging between 0 and 186, compared to >200 to non-outbreak strains [26, 28]. If more genomic data had been available, it is possible that shorter ADs within clusters would have been observed, providing stronger support for clustering.

## Conclusion

Finetype-based systematic cluster detection for IMD-B can identify additional potential clusters for further epidemiological and/or genomic investigations. However, the potential to prevent additional IMD-B cases is limited, mainly due to small cluster size and challenges of implementing public health measures when cases are scattered across non-specific community settings. Given the high strain diversity, monthly mapping of finetype within routine surveillance might be less resource-intensive while identifying the same clusters as SaTScan™ and may still be meaningful given IMD-B severity.

## Data Availability

Data cannot be shared due to privacy reasons.
